# Interleukin-like EMT inducer regulates partial phenotype switching in MITF-low melanoma cell lines

**DOI:** 10.1371/journal.pone.0177830

**Published:** 2017-05-17

**Authors:** Ken Noguchi, Annamarie C. Dalton, Breege V. Howley, Buckley J. McCall, Akihiro Yoshida, J. Alan Diehl, Philip H. Howe

**Affiliations:** 1 Department of Biochemistry and Molecular Biology, College of Medicine, Medical University of South Carolina, Charleston, SC, United States of America; 2 Hollings Cancer Center, Charleston, SC, United States of America; Medical College of Wisconsin, UNITED STATES

## Abstract

ILEI (*FAM3C*) is a secreted factor that contributes to the epithelial-to-mesenchymal transition (EMT), a cell biological process that confers metastatic properties to a tumor cell. Initially, we found that ILEI mRNA is highly expressed in melanoma metastases but not in primary tumors, suggesting that ILEI contributes to the malignant properties of melanoma. While melanoma is not an epithelial cell-derived tumor and does not undergo a traditional EMT, melanoma undergoes a similar process known as phenotype switching in which high (micropthalmia-related transcription factor) MITF expressing (MITF-high) proliferative cells switch to a low expressing (MITF-low) invasive state. We observed that MITF-high proliferative cells express low levels of ILEI (ILEI-low) and MITF-low invasive cells express high levels of ILEI (ILEI-high). We found that inducing phenotype switching towards the MITF-low invasive state increases ILEI mRNA expression, whereas phenotype switching towards the MITF-high proliferative state decreases ILEI mRNA expression. Next, we used *in vitro* assays to show that knockdown of ILEI attenuates invasive potential but not MITF expression or chemoresistance. Finally, we used gene expression analysis to show that ILEI regulates several genes involved in the MITF-low invasive phenotype including JARID1B, HIF-2α, and BDNF. Gene set enrichment analysis suggested that ILEI-regulated genes are enriched for JUN signaling, a known regulator of the MITF-low invasive phenotype. In conclusion, we demonstrate that phenotype switching regulates ILEI expression, and that ILEI regulates partial phenotype switching in MITF-low melanoma cell lines.

## Introduction

Malignant melanoma was considered an incurable disease until the advent of vemurafenib, a kinase inhibitor targeting *BRAF* V600E, and nivolumab, an immune checkpoint inhibitor targeting PD-1 [1–5]. While these drugs are considered to be breakthroughs, they are not without their faults. For instance, in the case of the vemurafenib many patients exhibit strong tumor debulking but most of the patients that respond rapidly develop relapsing tumors [6]. On the other hand, patients treated with nivolumab exhibit a durable response but few patients respond at all [2]. In order to build upon these breakthroughs, it is critical to develop a molecular understanding of why these therapies fail.

Phenotype switching is a phenomenon in which melanoma cell lines interconvert between a proliferative state and an invasive state, and is well recognized as a molecular mechanism for the failure of both kinase inhibitors and checkpoint inhibitors [7–13]. The primary driver of the proliferative state is MITF (micropthalmia-related transcription factor), which regulates melanocyte differentiation by activating the transcription of pigment producing genes such as *PMEL*, which encodes the premelanosome protein, and *TYR*, which encodes tyrosinase [14–18]. Conversely, the invasive state is classified by the lack of MITF because the master driver for this group is still unclear. The invasive cells express many genes related to neural crest biology including the non-canonical WNT ligand *WNT5A*, and the growth factor receptors *AXL* and *NGFR* (p75), and based on these gene targets TGF-β and JUN signaling have been proposed as regulators of the invasive phenotype [9, 10, 12, 19–21]. Phenotype switching was originally described based on gene expression data, which revealed that the transcriptomes of melanoma cell lines [22, 23] and patient samples [21] could be separated into distinct proliferative and invasive states. In addition to the finding that melanoma cells could be classified by these two descriptors, several groups have described that melanoma cells can reversibly switch between these two subsets either by direct genetic manipulation of MITF or environmental cues like TGF-β, inflammation, and hypoxia [17, 24–27]. Importantly, many aspects of the phenotype switch resemble the epithelial-to-mesenchymal transition (EMT) [22, 28].

EMT is a cell biological process in which epithelial cells with apical-basal polarity undergo cytoskeletal rearrangement to become motile mesenchymal cells. The transitioned mesenchymal cells have several qualities in addition to motility including the capacity to degrade basement membrane, survive in suspension, resist chemotherapy, and self-renew as cancer stem cells [29]. This behavioral change is coupled with molecular alternations including a cadherin switch from E- to N-cadherin, and also an activation of transcription factors such as Snail and ZEB [30–32]. EMT is clearly involved in cancer progression, but the exact contribution is much more complex than the reductionist model in which cells that undergo EMT are metastatic [29, 33–36]. There is evidence for a partial EMT or a hybrid E/M phenotype in which the cell blends epithelial and mesenchymal traits [29, 37–40]. For instance, knockdown of the EMT-inducing transcription factor PRRX1 induces an epithelial morphology along with a capacity for 3-D growth, which is classically ascribed to mesenchymal-like cells [41]. Compared to factors activating a full EMT those contributing to a partial EMT are poorly defined in the literature, even though the partial EMT state has been described as the primary driver of EMT-related pathology [39, 40].

In addition to pathology, EMT is known to contribute to various stages of embryonic development. Notably, it allows neural crest cells to delaminate from the neural tube and migrate through harsh conditions to colonize peripheral sites and differentiate into a wide variety of cell types [42]. It has been hypothesized by several groups that tumors originating from the neural crest, such as melanoma, may be hard-wired to re-activate the EMT machinery and thus more reliant on EMT during tumorigenesis [42–47].

EMT is regulated by many different stimuli including the cytokine TGF-β, which regulates EMT through transcription factors like ZEB or microRNAs such as miR-200 [32, 35, 48]. In addition to these classic EMT regulators, our group has described a post-transcriptional mechanism of TGF-β-induced EMT mediated through the translational repressor hnRNP-E1 [49–54]. Briefly, hnRNP-E1 inhibits the translation of EMT-specific mRNA molecules including the adaptor molecule DAB2 and the cytokine ILEI (Interleukin-like EMT inducer, FAM3C). Upon TGF-β stimulation, AKT2 phosphorylates hnRNP-E1 to dissolve the translational repressor complex, thus allowing for active translation of EMT-specific mRNA molecules. In this manuscript we will focus on one such target, ILEI.

FAM3C or ILEI was originally identified using a secondary structure-based prediction strategy to discover novel cytokines [55]. It was predicted that the FAM3 family of proteins would have secreted cytokine activity due to the presence of a four-helix-bundle commonly observed in the interleukin family of cytokines. Subsequently, ILEI has been described as an inducer of the epithelial-to-mesenchymal transition [56–60]. The only described regulators of ILEI are autophagy [61], the ubiquitin/proteasome system [62], and TGF-β/AKT2/hnRNP-E1 [49, 50, 57]. A major hindrance to the study of ILEI as a secreted molecule has been the challenge of producing a biologically active recombinant ILEI, due in part to the post-translational processing of ILEI and also the lack of a consistent biological readout for ILEI activity [57–59]. Therefore, a critical need to progress our molecular understanding of ILEI is the identification of transcriptional targets to use as markers of ILEI activity.

In the present study, we describe the contribution of ILEI to phenotype switching in melanoma. Initially we noted that *ILEI* mRNA expression is higher in melanoma metastases than primary tumors. We found in melanoma cell lines that ILEI is highly expressed in MITF-low invasive cells, and that phenotype switching between the MITF-low invasive state and the MITF-high proliferative state can alter ILEI expression. We demonstrate that ILEI depletion in cells attenuates their invasive potential but not MITF expression or chemoresistance, and through gene expression analysis identify novel ILEI transcriptional targets. Our results suggest that ILEI plays a role in phenotype switching in melanoma cells.

## Materials & methods

### Constructs

Lentiviral shRNAs were obtained from the MUSC Hollings Cancer Center shRNA Shared Resource Technology. All vectors used in this study are listed in S1 Table. MITF coding sequence overexpression vector was generated as follows: ApaI/EcoRI double digest was conducted on pCMV-Tag4A-MITF-M (wt), and ligated into the ApaI/EcoRI sites of pLenti-puro. pCMV-Tag4A-MITF-M (wt) was a generous gift from Yarden Samuels (Addgene plasmid #31151). pLenti-puro was a gift from Ie-Ming Shih (Addgene plasmid #39481) [63]. PTEN coding sequence overexpression vector was generated as follows: PTEN coding sequence was amplified from pCMV Flag WT-PTEN with BamHI and EcoRI sites by PCR, the PCR product was double digested by BamHI and EcoRI, and ligated into the BamHI/EcoRI sites of pLenti-puro. pCMV Flag WT-PTEN was a gift from Hong Wu (Addgene plasmid #22231) [64]. ILEI overexpression vector is the full coding sequence with a C terminal V5 tag in the pLX304 backbone, and this was purchased from DNASU Plasmid Repository (Tempe, AZ; deposited by David Root) [65]. The empty pLX304 backbone was a gift from David Root (Addgene plasmid # 25890) [65].

### Lentivirus production

Lentivirus was generated by seeding 293T (1 x 10^6^ cells; Takara Bio; Mountainview, CA; USA) to a 60 mm cell culture dish, and transfecting with 6 μL Lipofectamine 2000 (ThermoFisher; Waltham, MA; USA), 1 μg pLKO vector, 0.75 μg psPAX2, and 0.25 μg pMD2.G. 24h post-transfection the media was changed, and 48 and 72h post-transfection the media was harvested. Viral supernatant was cleared by centrifugation, filtered through 0.22 μm filter, and stored at -80° until use.

### Cell culture conditions

The following human melanoma cell lines were used: 501-Mel, Sk-Mel-28, WM3912, WM983B, WM793, 1205Lu, and WM9. These cell lines were purchased from ATCC, Coriell, or were a generous gift from Dr. J. Alan Diehl or Dr. Alain Mauviel. The following normal human melanocyte cell line was used: Primary Epidermal Melanocytes; Normal, Human, Neonatal. This cell line was purchased from ATCC (Manassas, VA; USA). All cell lines were cultured at 37°, 5% CO_2_ in RPMI-1640 (Hyclone; Logan, UT; USA) supplemented with 10% FBS (Atlanta Biologicals; Flowery Branch, GA; USA), Antibiotic-Antimycotic (100x; ThermoFisher; Waltham, MA; USA), and prophylactic plasmocin (InvivoGen; San Diego, CA; USA).

Stable cell lines were generated by lentiviral transduction with polybrene (8 μg/ml; Sigma-Aldrich; St. Louis, MO; USA). 24h post-transduction the media was changed, and 48h post-transduction the cells were selected and cultured with 0.125–0.5 μg/ml puromycin, 0.5–5 μg/ml blasticidin (InvivoGen; San Diego, CA; USA). Pools of stably transduced cells were analyzed.

Transient transfections were conducted with X-tremeGENE 9 DNA transfection reagent (Sigma-Aldrich; St. Louis, MO; USA) using 0.5 μg DNA and 1.5 μL X-tremeGENE 9 in 100 μL Opti-MEM I Reduced Serum Medium (ThermoFisher; Waltham, MA; USA) per 6 well plate. 24h post-transfection the media was changed, and 48h post-transfection the cells were harvested. siRNA transfections were conducted with Lipofectamine 3000 (ThermoFisher; Waltham, MA; USA) with 25 pmol siRNA and 7.5 μL Lipofectamine RNAiMAX in 250 μL Opti-MEM I Reduced Serum Medium (ThermoFisher; Waltham, MA; USA) per 6 well plate. 24h post-transfections the media was changed, and 72h post-transfection the cells were harvested. siRNA molecules used in this study are listed in S1 Table.

### Chemicals

Vemurafenib and LY-294002 were purchased from Selleckchem (Houston, TX; USA). Chloroquine and MG-132 were purchased from Sigma-Aldrich (St. Louis, MO; USA). All compounds were stored in DMSO at -20°. TGF-β2 was a generous gift from Genzyme Corporation (Cambridge, MA; USA).

### Immunoblot analysis

Whole cell lysates were extracted as follows: 100 μL of Tris-Triton lysis buffer (20 mM Tris pH 7.5, 1% Triton X-100, 10% glycerol, 137 mM NaCl, 2 mM EDTA, and Halt Protease and Phosphatase Inhibitor cocktail [ThermoFisher; Waltham, MA; USA]) was added to a 6 well cell plate, cells were immediately scraped, incubated on ice for 30 minutes, and cleared by centrifugation for 20 minutes at 16,000 x g. Protein concentrations were measured with Bradford Protein Assay (BioRad; Hercules, CA; USA). For conditioned medium immunoblots, cells were serum starved in RPMI/0% FBS overnight, medium was harvested, and precipitated using trichloroacetic acid/acetone. Protein samples were denatured by incubating at 95° for 5 minutes with 1x Laemmli reducing denaturing sample buffer (60 mM Tris-Cl pH 6.8, 1% SDS, 10% glycerol, 5% BME). 1–30 μg of whole cell lysate was resolved on an 8, 10, or 12% polyacrylamide SDS gel, and transferred onto PVDF membrane. Membranes were blocked for 1h at RT in 5% skim milk/Tris-buffered saline with 0.01% Tween-20 (TBST) and incubated overnight at 4° on primary antibody + 5% skim milk/TBST. The following primary antibodies were used: ILEI (ab72182; Abcam; Cambridge, MA; USA; 1:1,000), MITF (ab12039; Abcam; Cambridge, MA; USA; 1:1,000) α-tubulin (2144; Cell Signaling; Danvers, MA; USA; 1:10,000), Total AKT (4691; Cell Signaling; Danvers, MA; USA; 1:1,000), p-AKT S473 (4060; Cell Signaling; Danvers, MA; USA; 1:1,000), p-AKT T308 (13038; Cell Signaling; Danvers, MA; USA; 1:1,000), p-ERK T202/Y204 (4370; Cell Signaling; Danvers, MA; USA; 1:2,000), Total ERK (9120; Cell Signaling; Danvers, MA; USA; 1:1,000), PTEN (#9188; Cell Signaling; Danvers, MA; USA; 1:1,000), LC3B (#2775; Cell Signaling; Danvers, MA; USA; 1:1,000), BIM (#2933; Cell Signaling; Danvers, MA; USA; 1:1,000), V5 (R960; ThermoFisher; Waltham, MA; USA; 1:1,000), GAPDH (sc-32233; Santa Cruz; Dallas, TX; USA; 1:10,000), HSP90 (sc-13119; Santa Cruz; Dallas, TX; USA; 1:10,000), Ubiquitin (sc-8017; Santa Cruz; Dallas, TX; USA; 1:200). After primary antibody incubation, membranes were washed 4x 15 minutes in TBST and incubated for 1h at RT on secondary antibody + TBST. The following secondary antibodies were used: Goat anti-Mouse IgG (31430; ThermoFisher; Waltham, MA; USA; 1:10,000) and Goat anti-Rabbit IgG (31460; ThermoFisher; Waltham, MA; USA; 1:10,000). After secondary antibody incubation, membranes were washed 4x 15 minutes in TBST and detected using Luminata Forte Western HRP substrate (EMD Millipore; Darmstadt, Germany) and HyBlot CL Autoradiography Film (Denville; Holliston, MA; USA) or CCD camera (BioRad ChemiDoc System; BioRad, Hercules, CA, USA).

### PCR analysis

Total RNA was isolated using Trizol (Thermo Fisher Scientific; Waltham, MA; USA). Reverse transcription was performed using oligo dT primers and M-MuLV Reverse Transcriptase (New England BioLabs, Ipswitch, MA; USA). Semi-quantitative PCR was conducted on 10 ng of cDNA using Maxima Hot Start PCR Master Mix (Thermo Fisher Scientific; Waltham, MA; USA). Real-time quantitative PCR was conducted using iQ SYBR Green Supermix (BioRad; Hercules, CA; USA) using CFX384 Real-Time System (BioRad; Hercules, CA; USA). Reactions were conducted on 50 pg—10 ng cDNA. Primers are listed in S2 Table. Relative gene expression was calculated using RFX Manager software, and genes were normalized to GAPDH internal control.

### ELISA

Cells were serum starved in RPMI/0% FBS overnight, and medium was harvested and analyzed using FAM3C ELISA kit (MyBioSource; San Diego, CA; USA).

### Cell proliferation assays

Cells (1 x 10^5^, 1 ml of complete medium) were seeded in a 6 well plate, and every 24h cells were harvested with trypsin and manually counted using a hemocytometer.

### Wound healing assays

Cells (3 x 10^5^, 1 ml of complete medium) were seeded in a 24 well plate, and a 1 ml pipette tip was used to scratch the cells. Images were recorded from 0 to 24 hours, and analyzed using ImageJ (NIH; Bethesda, MD; USA).

### Transwell migration assays

Cells (5 x 10^4^, 0.05 ml of RPMI/0.1% BSA) were seeded in the upper chamber of a 24 well Transwell Clear Polyester Membrane Inserts (Corning; Corning, NY; USA). 0.5 ml of complete medium was added to the lower chamber. After 24h cells were fixed in 70% ethanol and stained in 0.2% crystal violet.

### Transwell invasion assays

Cells (5 x 10^4^, 0.1 ml in RPMI/0.1% BSA) were seeded in the upper chamber of a 24 well BD BioCoat Matrigel Invasion Chambers (BD Biosciences; San Jose, CA). 0.5 ml of complete medium was added to the lower chamber. After 24h, cells were fixed in methanol and stained in 0.5% crystal violet/20% methanol [53].

### 3-D invasion assays

Cells (5 x 10^3^, 0.05 ml of Essential 8 Medium [Thermo Fisher Scientific; Waltham, MA; USA]) were seeded in a 96 well ultra-low attachment spheroid microplate (Corning; Corning, NY; USA). After 48h, 0.05 ml of Cultrex 3D Spheroid Invasion Matrix (Trevigen; Gaithersburg, MD; USA) was added and incubated for 1h at 37°. 0.1 ml of complete medium was layered on top of the invasion matrix. The spheroids were imaged after 72h using a Leica microscope, Amscope camera, and AmscopeX software.

### MTT assays

Cells (2 x 10^3^, 0.2 ml complete medium) were seeded in a 96 well plate. After 24h, cells were treated with drug as indicated. After 72h cells were treated for 3h with 10 μL of MTT solution (3–4,5-Dimethylthiazol-2-yl)-2,5-diphenyltetrazolium bromide, 5 mg/ml; purchased from Sigma-Aldrich; St. Louis, MO; USA), and then treated for 1h with 100 μL of MTT stop solution (40% dimethyl formamide and 20% sodium dodecyl sulfate). Absorbance was read at 570 nm using a Wallac plate reader.

### Clonogenic assay

Cells (1–5 x 10^4^, 1 ml of complete medium) were seeded in a 10 cm plate. After 24h cells were treated with drug as indicated. After 7d cells were fixed in 3.7% PFA (Sigma-Aldrich; St. Louis, MO; USA) and stained with 0.2% crystal violet/20% methanol.

### FACS analysis

Cells (1 x 10^6^, 5 ml complete medium) were seeded in 6 cm dishes. After 24h cells were treated with DMSO vehicle or 5 μM vemurafenib (BRAFi) for 48h. Then, cells were harvested using trypsin and analyzed using FITC Annexin V Apoptosis Detection Kit I (#556547; BD Bioscience; San Jose, CA; USA).

### Microarray analysis

Total RNA isolated using Trizol (Thermo Fisher Scientific; Waltham, MA; USA) was sent for microarray analysis at the MUSC ProteoGenomics Facility. Labeling was conducted using 3’ IVT Plus kit. GeneChip PrimeView Human Gene Expression Array was used (Affymetrix; Santa Clara, CA; USA). Data was analyzed using Gene Set Enrichment Analysis [66, 67]. Heatmap was made using ClustVis [68]. The raw data was deposited to NCBI Gene Expression Omnibus database under series accession number GSE95509. Fold-change was calculated by the average of shSCR/shILEI in both WM9 and 1205Lu cells.

### Databases

Data on ILEI IHC comparing melanoma and breast cancer tissue was obtained from the Human Protein Atlas [69]. Data on ILEI expression compared to MITF expression was obtained from cBioPortal [70]. Data on ILEI expression in primary melanoma compared to metastatic melanoma was obtained from GEO [71, 72].

### Statistical analyses

Data are mean +/- standard deviation unless indicated otherwise. p < 0.05 by unpaired Student’s T-test is considered significant. Representative experiments are repeated at least twice.

## Results

### ILEI expression in melanoma (Fig 1)

**Fig 1:**
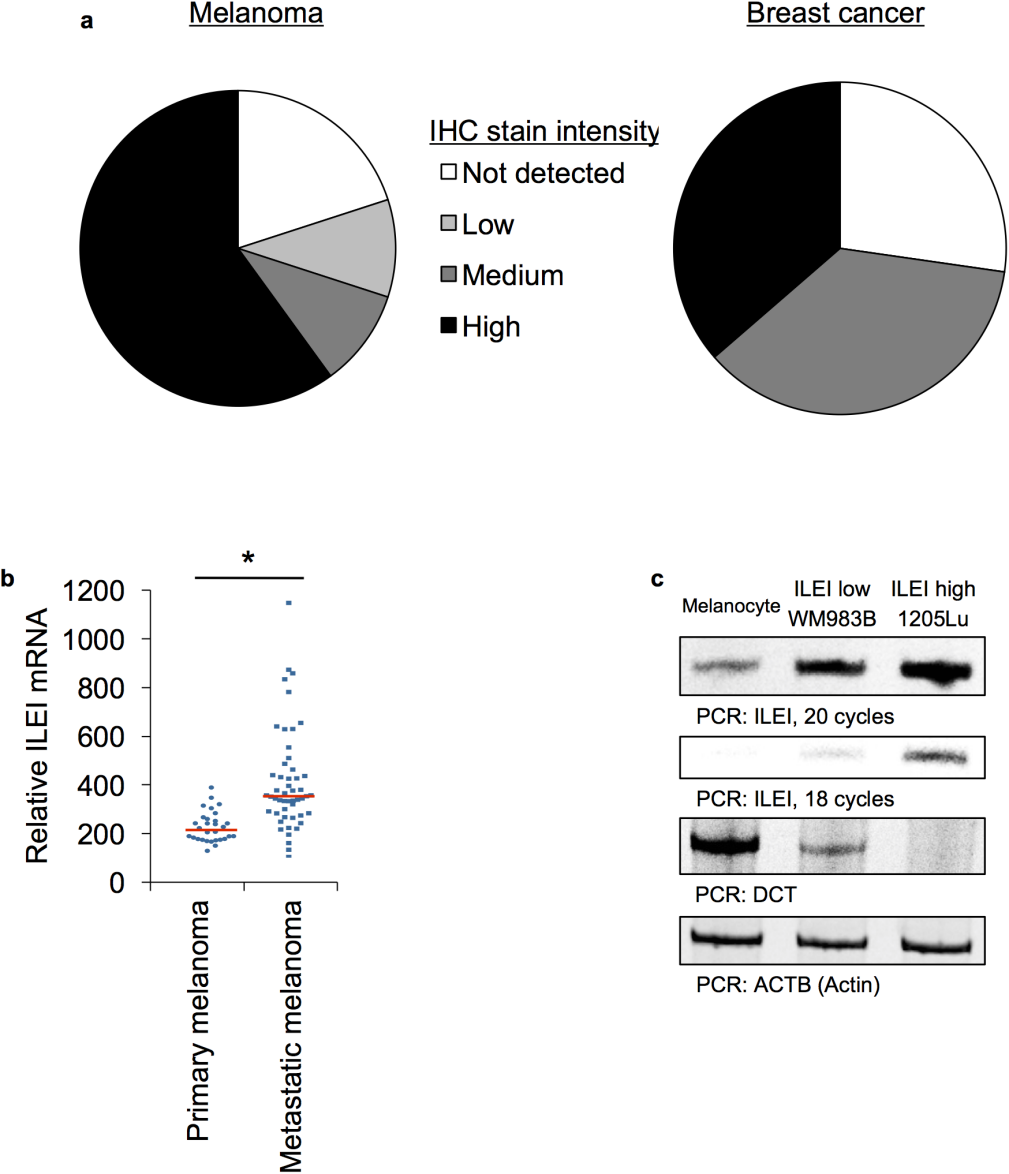
ILEI expression in melanoma. **A.** Data from the Human Protein Atlas showing IHC stain intensity for ILEI in melanoma vs breast cancer [69]. **B.** Data from NCBI GEO database [72] comparing ILEI mRNA levels in primary vs metastatic melanoma patient samples, accession GSE8401, N_primary_ = 31, N_metastatic_ = 52, mean_primary_ = 136, mean_metastatic_ = 323, SD_primary_ = 63, SD_metastatic_ = 208, p = 5 x 10^−6^ by unpaired Student’s t-test [72]. **C.** Semi-quantitative RT-PCR analysis of ILEI and DCT in primary epidermal melanocytes, WM983, and 1205Lu cells.

We used the Human Protein Atlas database and found that melanoma, when compared to breast cancer, expressed high levels of ILEI (Fig 1A) [69]. We chose to compare melanoma with breast cancer because ILEI has been traditionally studied in the breast cancer model. Second, we found using the GEO database that ILEI mRNA expression is higher in melanoma metastases when compared to primary tumors (Fig 1B) [71, 72]. We confirmed these findings by conducting RT-PCR analysis of normal melanocytes, the poorly metastatic WM983B, and the highly metastatic 1205Lu cell lines, and found that ILEI expression increased with aggressiveness (Fig 1C). As a surrogate measure for aggressiveness we have used dopachrome tautomerase (*DCT*), which is an MITF-target gene. MITF and its target genes are known to decrease as melanomas gain invasive capacity, and we observed an increase in ILEI along with a decrease in DCT. Based on these initial findings, we hypothesize that ILEI mRNA expression contributes to the malignant properties of melanoma.

### ILEI expression in melanoma cell lines (Fig 2, S1 Fig)

**Fig 2:**
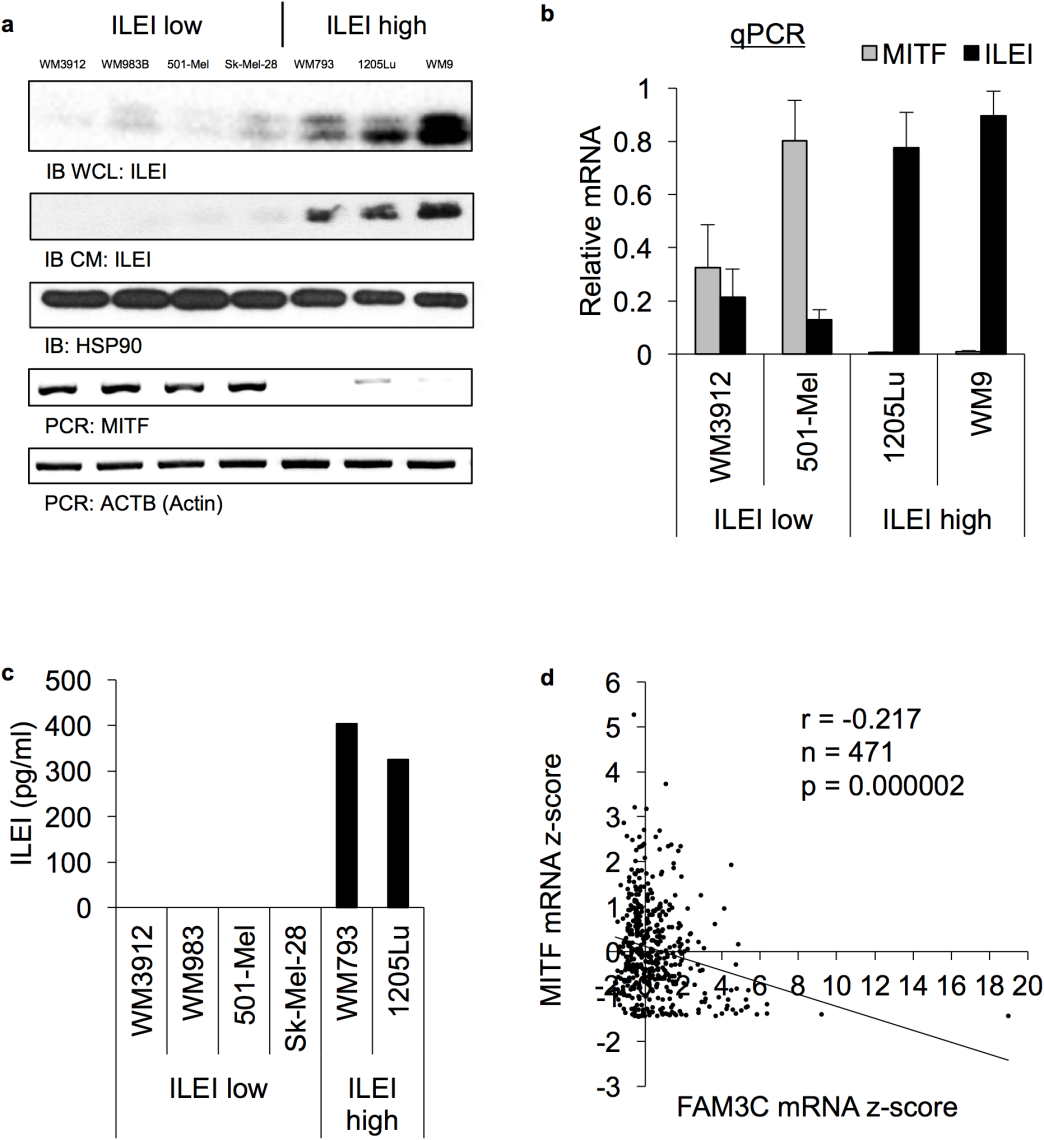
ILEI expression in melanoma cell lines. **A.** Immunoblot and semi-quantitative RT-PCR analysis of ILEI and MITF levels in WM3912, WM983B, 501-Mel, Sk-Mel-28, WM793, 1205Lu, and WM9 cells. IB WCL indicates ILEI in the whole cell lysate, whereas IB CM indicates ILEI in protein that was precipitated out of serum-free conditioned medium. **B.** Quantitative RT-PCR analysis of MITF and ILEI levels in WM3912, 501-Mel, 1205Lu, and WM9 cells. Grey bars indicate MITF mRNA and black bars indicate ILEI mRNA. N = 3, mean +/- SD, transcript levels normalized to GAPDH. **C.** ILEI ELISA of conditioned medium from WM3912, WM983B, 501-Mel, Sk-Mel-28, WM793, and 1205Lu cells. Prior to conditioned medium analysis, the cells were cultured for 24h in serum-free RPMI. **D.** Melanoma patient RNA-seq data from cBioPortal [70, 73] showing MITF mRNA z-score vs FAM3C mRNA z-score in melanoma patient samples. The correlation was calculated using Pearson’s correlation coefficient: r = -0.217, N = 471, p = 1.94 x 10^−6^.

We used immunoblot, PCR, and ELISA analysis ILEI expression in a panel of melanoma cell lines and observed two distinct populations of cells either expressing low (ILEI-low) or high (ILEI-high) levels of ILEI (Fig 2A–2C). We characterized the ILEI antibody (Abcam, ab72182) by conducting immunoblot analysis of ILEI knockdown cell lines. The intracellular form of ILEI ran as a doublet between 20 and 25 kDa, whereas secreted ILEI ran at 20 kDa (S1 Fig and data not shown). We noted that the ILEI-low population correlated with the MITF-high proliferative cells whereas the ILEI-high population correlated with the MITF-low invasive cell lines (Fig 2A–2C). Further, we used TCGA RNA-seq data from cBioPortal to confirm the negative correlation (Pearson’s correlation coefficient: r = -0.217, N = 471, p = 1.94 x 10^−6^) between ILEI and MITF in melanoma patient samples (Fig 2D) [70, 73, 74]. Based on these initial findings, we hypothesized that ILEI is regulated during the phenotype switch.

### The effect of phenotype switching on ILEI expression (Figs 3 and S2)

In order to test the effect of phenotype switching on ILEI expression we used two different models: 1) addition of exogenous stimuli to induce a natural phenotype switch between the MITF-high proliferative and the MITF-low invasive phenotype; and, 2) directly modulate MITF levels to force the phenotype switch.

**Fig 3:**
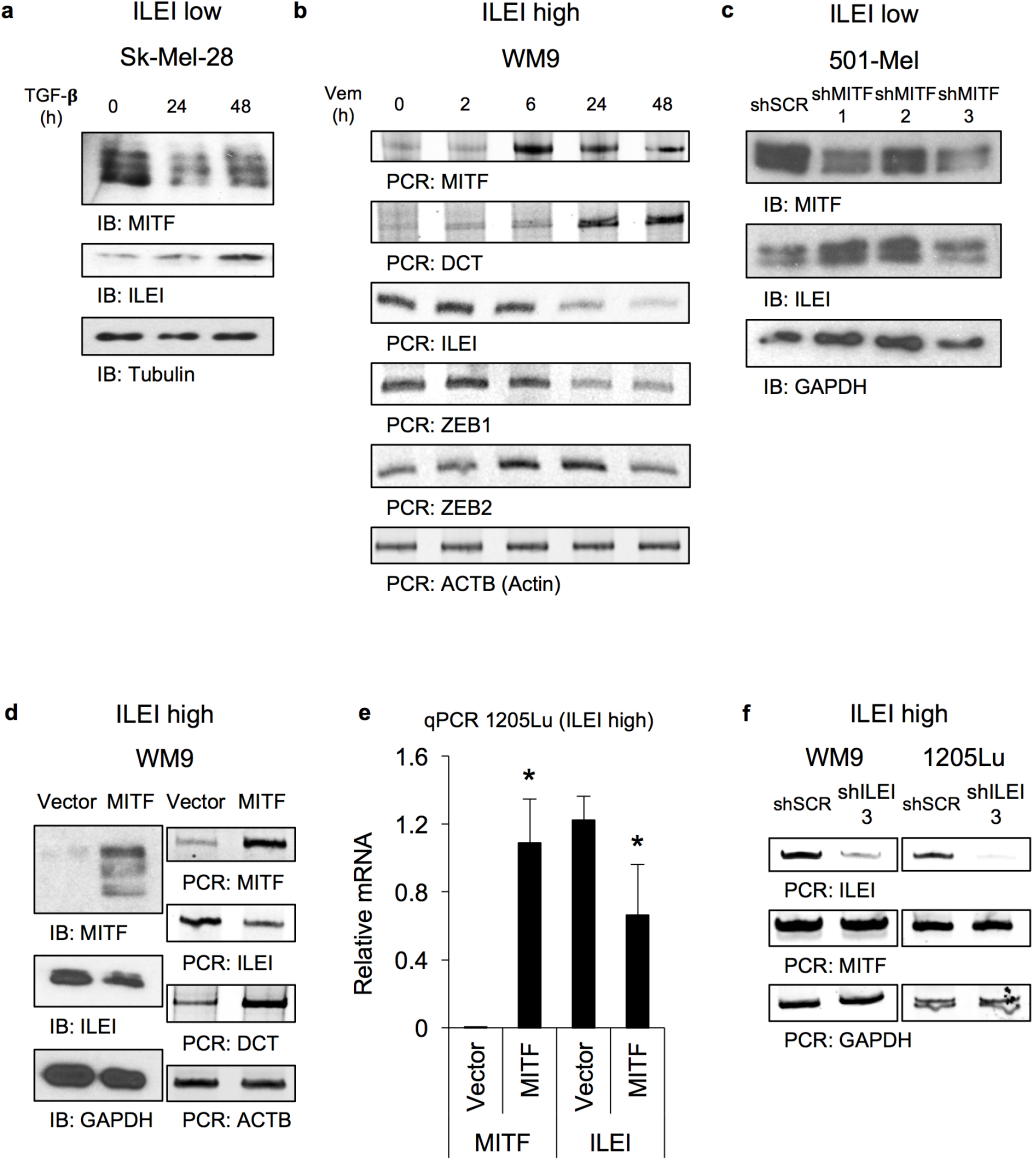
The effect of phenotype switching on ILEI expression. **A.** Immunoblot analysis of MITF and ILEI levels in ILEI-low Sk-Mel-28 cells treated with TGF-β (0 to 48 h, 5 ng/ml). **B.** Semi-quantitative RT-PCR analysis of MITF, DCT, ILEI, ZEB1, ZEB2, and ACTB levels in ILEI-high WM9 cells treated with vemurafenib (BRAFi, 0 to 48 h, 1 μM). **C.** Immunoblot analysis of MITF and ILEI levels in ILEI-low 501-Mel stably expressing scrambled shRNA or three independent shRNA hairpins specific for MITF. **D.** Immunoblot and semi-quantitative RT-PCR analysis of MITF, ILEI, and DCT levels in WM9 stably expressing empty vector or MITF. **E.** Quantitative RT-PCR analysis of MITF and ILEI levels in 1205Lu cells transiently expressing empty vector or MITF. N = 3, mean +/- SD, mRNA levels normalized to GAPDH, * indicates p < 0.05 by unpaired Student’s t-test. **F.** Semi-quantitative RT-PCR analysis of MITF and ILEI levels in WM9 and 1205Lu cells stably expressing scrambled shRNA or shRNA targeting ILEI.

In the first instance, we used either TGF-β, which induces the invasive phenotype, or the BRAF inhibitor vemurafenib, which can induce the proliferative phenotype in certain cell lines [9, 25, 28, 75, 76]. When we treated MITF-high ILEI-low Sk-Mel-28 cells with TGF-β we observed the expected decrease of MITF along with an increase in ILEI expression (Fig 3A). Similar results were seen at the mRNA level using semi-quantitative PCR (data not shown). Conversely, when we treated MITF-low ILEI-high WM9 cells with vemurafenib we observed the expected increase in MITF and DCT. The increase in DCT, which is a MITF target gene, suggests that the increase in MITF mRNA is functional. We also observed a decrease in ILEI expression (Fig 3B). In addition, we confirmed that vemurafenib downregulates the invasive melanoma marker ZEB1, and upregulates the proliferative melanoma marker ZEB2.

Next, we transduced MITF-high ILEI-low cells with three independent shRNA hairpins specific for MITF and used immunoblot analysis to observe a decrease in MITF and an increase in ILEI (Fig 3C). We also conducted the MITF overexpression experiments in MITF-low ILEI-high cells, and observed the expected increase in MITF and DCT along with a decrease in ILEI (Fig 3D–3E). These data not only suggest that MITF regulates ILEI during phenotype switching, but that ILEI might also regulate MITF. To test this possibility, we transduced MITF-low ILEI-high cells with shRNA specific for ILEI. We did not observe any change in MITF expression but nevertheless wished to determine whether ILEI contributes to other aspects of phenotype switching.

### Other regulators of ILEI expression (S2 Fig)

AKT signaling, high autophagy, and inhibition of proteasomal degradation have been reported to promote ILEI expression [49, 50, 61, 62]. We wanted to know if any of these other factors could regulate ILEI expression in our system so we began by testing AKT signaling. We found that ILEI high cells were low for PTEN, an inhibitor of AKT signaling, and high for phosphorylated AKT. However, when we knocked down PTEN with two independent shRNA hairpins we did not see any change in ILEI expression (S2A and S2B Fig). These results suggested that AKT signaling did not contribute to ILEI expression in melanoma cell lines.

Next, we tested the contribution of autophagy to ILEI expression. We measured autophagic flux by using chloroquine (CQ) treatment to inhibit lysosomal degradation followed by immunoblot analysis for LC3B (Microtubule-associated proteins 1A/1B light chain 3B), a known target of autophagic degradation. CQ-mediated inhibition of lysosomal degradation allows accumulation of LC3B and the relative accumulation is considered a measure of active autophagy or autophagic flux [77]. We found that CQ treatment increased LC3B in ILEI-low cells more than ILEI-high cells, which suggested that ILEI-low cells had higher autophagic flux (S2C Fig). This finding is consistent with ILEI-low cells expressing high PTEN, which is a positive regulator of autophagy (S2C Fig).

Finally, it has been shown that ILEI can be degraded by the ubiquitin proteasome system [62]. Thus, we treated cells with MG-132 to inhibit proteasomal degradation. We confirmed the efficacy of the MG treatment by observing an increase in a high molecular weight ubiquitin smear, which suggested an accumulation of ubiquitinated substrates (S2C Fig). However, we did not see any change in ILEI, suggesting that ILEI is not degraded by the proteasome in melanoma cell lines (S2C Fig).

### The effect of ILEI knockdown on phenotype switching (Figs 4, S3 and S4)

**Fig 4:**
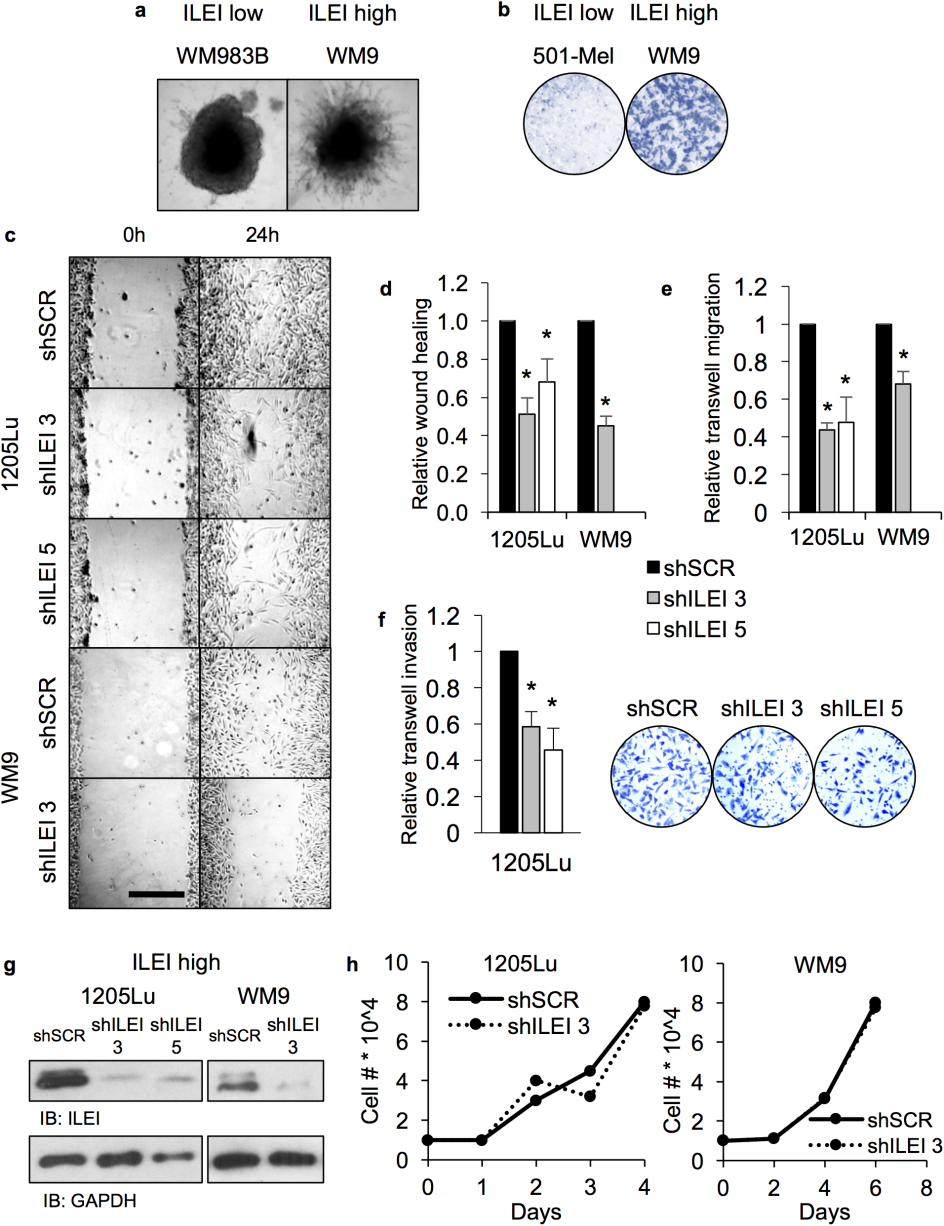
The effect of ILEI knockdown in ILEI-high/MITF-low cells on invasion and proliferation. **A.** 3-D spheroid invasion assay of ILEI-low WM983B or ILEI-high WM9 cells in extracellular matrix. **B.** Transwell migration assay of ILEI-low 501-Mel or ILEI-high WM9 cells. Images are pseudo colored blue. **C.** Wound healing assay of 1205Lu or WM9 expressing shSCR, shILEI 3, or shILEI 5. Images shown are representative of three independently seeded replicates. **D.** Quantification of panel E using ImageJ software. N = 3, mean +/- SEM, and * indicates p < 0.05 by unpaired Student’s t-test. Solid bars indicate shSCR, grey bars indicate shILEI 3, and white bars indicate shILEI 5. **E.** Transwell migration assay of 1205Lu or WM9 expressing shSCR, shILEI 3, or shILEI 5. N = 3, mean +/- SEM, and * indicates p < 0.05 by unpaired Student’s t-test. **F.** Transwell invasion assay of 1205Lu expressing shSCR, shILEI 3, or shILEI 5. N = 3, mean +/- SEM, and * indicates p < 0.05 by unpaired Student’s t-test. Images are representative of three independently seeded replicates, and pseudo colored in blue. **G.** Immunoblot analysis of ILEI-high 1205Lu or WM9 expressing shSCR, shILEI 3, or shILEI 5. **H.** Cell counts of 1205Lu or WM9 expressing shSCR or shILEI 3. Solid lines indicate shSCR and dotted lines indicate shILEI 3. Data is representative of three independently seeded replicates.

In order to assess the contribution of ILEI to MITF-independent aspects of phenotype switching we chose to investigate two well-described characteristics of phenotype switching; namely, invasion and chemoresistance [9, 12, 22, 24, 78–80]. Previous studies have shown that MITF-low cells reside at the leading edge of a tumor allowing for dissemination from the primary site [28]. Our results thus far indicate that MITF-low cells have high ILEI expression, thus we started by confirming that the ILEI-high cells used in this study are more invasive than the ILEI-low cells. We used both 3-D invasion and transwell migration assays to confirm that ILEI-high cells were more invasive than ILEI-low cells (Fig 4A and 4B). Next, we generated ILEI knockdown cells (two independent hairpins in 1205Lu and one hairpin in WM9 cells) to test invasive potential *in vitro*. We used several techniques (wound healing, transwell migration, and transwell invasion) and found that ILEI knockdown attenuates migration/invasion (Fig 4C–4F). We confirmed these results with the following control experiments: ILEI immunoblot to test the extent of the knockdown, and proliferation rate, which can confound migration/invasion experiments. In both experiments we saw the expected result that ILEI was indeed knocked down, and that ILEI knockdown did not affect the proliferation rate (Fig 4G and 4H).

In addition to invasion, MITF is also associated with chemoresistance and it has been shown that: 1) Cell lines that are more intrinsically resistant to vemurafenib (BRAFi) are MITF-low, and 2) Long-term treatment of cell lines with vemurafenib can lead to an invasive MITF-low population [8, 26]. Thus, we postulated that ILEI knockdown might decrease chemoresistance or that ILEI expression would increase upon acquisition of resistance. First, we confirmed that our MITF-low ILEI-high cells have higher intrinsic resistance to vemurafenib by treating cells with vemurafenib and conducting immunoblot analysis for the apoptosis marker BIM, MTT, or clonogenic assay (S3A–S3C Fig). Next, we used our ILEI knockdown cells and found that ILEI knockdown has a subtle effect on BIM induction and cell survival as measured by MTT assay, but has no effect on cell survival as measured by clonogenic assay or FACS analysis for the apoptosis markers Annexin V and PI (S3D–S3G Fig). From these studies we concluded that loss of ILEI does not affect vemurafenib resistance. An alternate possibility for ILEI contribution to chemoresistance was that long-term treatment of melanoma cells with vemurafenib would generate vemurafenib resistant cell lines with increased expression of ILEI. We confirmed that our cells were resistant to vemurafenib by treating either parent or long-term vemurafenib treated resistant cells with vemurafenib and measuring the induction of BIM (S4A Fig). According to our expectation vemurafenib-induced BIM was attenuated in the resistant cells, but to our surprise ILEI expression was either unaffected or decreased upon acquisition of resistance. From these studies, we conclude that ILEI knockdown decreases invasive potential but does not affect chemoresistance.

### The effect of ILEI overexpression on phenotype switching (S5 Fig)

Considering the effect of ILEI knockdown on ILEI-high cells, we also wanted to know the effect of ILEI overexpression on ILEI low cells. We generated MITF-high ILEI-low cells that overexpressed ILEI as seen by immunoblot of either the whole cell lysate or the conditioned medium (S5A Fig). Next, we tested the effects of ILEI overexpression on ILEI phenotype switching. We found that ILEI overexpression did not affect proliferation rate or wound healing (S5B and S5C Fig). While we observed a subtle effect of ILEI overexpression repressing vemurafenib-induced BIM induction, we saw by MTT assay that the survival of the cells was unaffected (S5D and S5E Fig). From these studies we conclude that ILEI overexpression does not affect phenotype switching.

### The effect of ILEI knockdown on gene expression (Fig 5; Tables 1 and 2)

**Fig 5:**
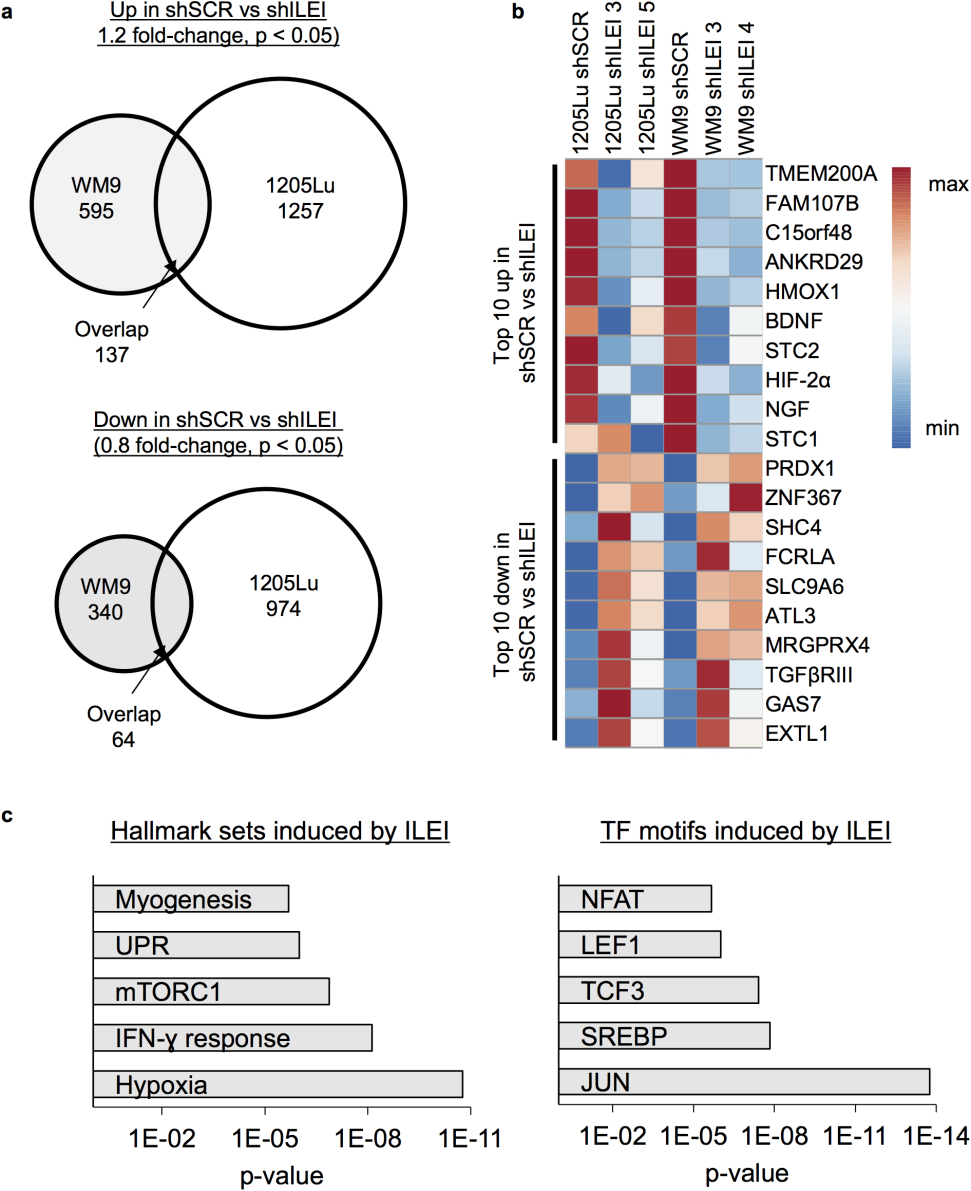
The effect of ILEI knockdown in ILEI-high/MITF-low cells on gene expression. **A.** Microarray analysis of ILEI-high 1205Lu or WM9 stably expressing shSCR, shILEI 3, shILEI 4, or shILEI 5. Venn diagrams represent numbers of genes up (1.2-fold) or down (0.8-fold) in shSCR vs shILEI (p < 0.05 in shSCR vs shILEI sample by unpaired Student’s t-test, N = 3). **B.** Heat map of top 10 genes up or down in shSCR vs shILEI (p < 0.05 in shSCR vs shILEI by unpaired Student’s t-test, N = 3). Heat map was generated using ClustVis [68]. **C.** Gene set enrichment analysis [66, 67] of genes up in shSCR vs shILEI (>1.2-fold and p < 0.05; 137 genes). Enriched hallmark pathways and transcription factor motifs are shown.

Since we observed a biological effect of ILEI knockdown in MITF-low ILEI-high cells we knew that ILEI was functional in these cells, and thus we conducted gene expression analysis on these cells to elucidate the molecular mechanism(s) of ILEI-modulated invasion. We conducted microarray analysis on two cell lines 1205Lu and WM9, stably transduced with shSCR, shILEI 3, shILEI 4, or shILEI 5 (S3 Table). Analysis of genes commonly regulated in both cell lines across all shRNA hairpins revealed a set of 137 genes up-regulated in shSCR vs shILEI and 64 genes downregulated in shSCR vs shILEI (Fig 5A). The top regulated genes included many that are involved in phenotype switching including CDH13, HIF-2α, and BDNF (Fig 5B; Tables 1 and 2) [23]. Interestingly, other well-characterized markers of phenotype switching including EMT transcription factors like ZEB1 and ZEB2 and invasive phenotype markers like WNT5A, AXL, and NGFR were not regulated across both cell lines (data not shown). We conducted gene set enrichment analysis on the genes that were up in shSCR vs shILEI and observed enrichment of genes regulated by hypoxia, mTOR signaling, and UPR, as well as JUN and NFAT transcription factor motifs known to regulate the invasive phenotype (Fig 5C) [66, 67]. Genes that were down in shSCR vs shILEI were not enriched for any gene sets (data not shown). Overall these data suggest that knockdown of ILEI in ILEI-high cells regulates the expression of genes that are important to the invasive MITF-low phenotype.

**Table 1:** Genes up in shSCR vs shILEI.

Gene Title	Gene Symbol	Fold-change
Chromosome 15 open reading frame 48	C15orf48	3.70
Stanniocalcin 2	STC2	2.78
Ferritin, heavy polypeptide 1	FTH1	2.52
Aldo-keto reductase family 1, member B1	AKR1B1	2.37
Heme oxygenase (decycling) 1	HMOX1	2.14
Endothelial PAS domain protein 1 (HIF-2α)	EPAS1	2.11
Sterile alpha motif domain containing 9-like	SAMD9L	2.11
Laminin, beta 3	LAMB3	2.05
Ankyrin repeat domain 29	ANKRD29	1.90
Transmembrane protein 200A	TMEM200A	1.86
Leucine rich adaptor protein 1-like	LURAP1L	1.84
DNA-damage-inducible transcript 3 (CHOP)	DDIT3	1.83
Adenosine monophosphate deaminase 3	AMPD3	1.80
Solute carrier family 38, member 1	SLC38A1	1.78
Lysine (K)-specific demethylase 5B (JARID1B)	KDM5B	1.77
Brain-derived neurotrophic factor	BDNF	1.76
Laccase (multicopper oxidoreductase) domain containing 1	LACC1	1.67
Raftlin, lipid raft linker 1	RFTN1	1.67
Nuclear receptor coactivator 3	NCOA3	1.65
Nuclear receptor coactivator 7	NCOA7	1.63
Tetratricopeptide repeat domain 17	TTC17	1.61
Vascular endothelial growth factor A	VEGFA	1.56
Aldo-keto reductase family 1, member B10 (aldose reductase)	AKR1B10	1.52
ERO1-like beta (S. cerevisiae)	ERO1LB	1.52
Glutamate-cysteine ligase, modifier subunit	GCLM	1.51
Thioredoxin reductase 1	TXNRD1	1.51
Adaptor-related protein complex 2, alpha 2 subunit	AP2A2	1.51
Interleukin 31 receptor A	IL31RA	1.50

All genes are significantly up in shSCR vs shILEI (1.5-fold, p < 0.05 by unpaired Student’s t-test) in both 1205Lu and WM9 cells.

**Table 2:** Genes down in shSCR vs shILEI.

Gene Title	Gene Symbol	Fold-change
Peroxiredoxin 1	PRDX1	0.08
Zinc finger protein 367	ZNF367	0.39
Solute carrier family 9, subfamily A	SLC9A6	0.44
SHC (Src homology 2 domain containing) family, member 4	SHC4	0.46
Fc receptor-like A	FCRLA	0.54
Transforming growth factor, beta receptor III	TGFBR3	0.57
Growth arrest-specific 7	GAS7	0.58
Exostoses (multiple)-like 1	EXTL1	0.59
Atlastin GTPase 3	ATL3	0.60
Fc receptor-like A	FCRLA	0.60
MAS-related GPR, member X3	MRGPRX3	0.62
SRY (sex determining region Y)-box 2	SOX2	0.64

All genes are significantly down in shSCR vs shILEI (0.66-fold, p < 0.05 by unpaired Student’s t-test) in both 1205Lu and WM9 cells.

## Discussion

Classical EMT factors are increasingly gaining recognition as regulators of neural crest-derived tumors such as melanoma. Herein we characterize the EMT-inducing cytokine ILEI in melanoma. We found that ILEI is highly expressed in MITF-low invasive melanoma cells, and that phenotype switch alters ILEI expression. Additionally, we found that ILEI knockdown in MITF-low cells attenuates invasive potential but does not affect MITF expression, or chemoresistance. Finally, we conducted gene expression analysis and show that ILEI regulates genes that are important to the MITF-low invasive phenotype.

With regards to phenotype switching and EMT, we offer the following discussion. EMT/phenotype switching encompasses a vast range of phenotypes including motility, resistance to apoptosis, and self-renewal and it is becoming increasingly clear that EMT is not a simple on/off switch but a long gradient with multiple paths between the epithelial and mesenchymal states. Consistent with this sort of gradient within EMT, there must be EMT factors that induce parts but not all of the EMT-phenotype [37, 39, 41]. There is obvious clinical relevance to this nuance given the evidence that the partial EMT state represents a more aggressive state of the tumor [29, 38, 40]. However, in the field of melanoma and phenotype switching there has not been a clear identification of factors involved in a partial phenotype switch. Previous gene expression analyses on patient samples or cell lines that define the phenotype switch identify a proliferative phenotype and an invasive phenotype, but the data also contain an overlooked intermediate state in-between the proliferative and invasive phenotypes [21, 22]. More recently, single-cell gene expression analyses have confirmed the presence of wide heterogeneity within a cell line or a tumor sample that is broadly categorized as proliferative of invasive [81, 82]. While it is clear that MITF is a major regulator of phenotype switching, there is also evidence for the existence of MITF-independent phenotype switching mechanisms. For instance, a recent paper showed that induction of ATF4 in melanoma cell lines inhibits MITF expression, but does not affect invasive potential [83]. Additionally, circulating tumor cell (CTC) studies have shown that melanoma CTCs express MITF, suggesting MITF-expressing cells can be actively invasive. [84, 85]

Based on the results of this study we speculate that phenotype switching regulates ILEI expression, but that ILEI contributes to a partial phenotype switch with other factors being required to drive a full phenotype switch. The first part of this statement, that phenotype switching regulates ILEI is based on the data that ILEI expression correlates with the phenotype of the cell (Fig 2) and that phenotype switching between the proliferative and invasive states alters ILEI expression (Fig 3). The second part of the statement that ILEI drives only a partial phenotype switch is based on the data that modulation of ILEI in MITF-low invasive cells attenuates invasive potential (Fig 4). However, ILEI does not modulate other aspects of phenotype switching including MITF and EMT-TF expression and chemoresistance (Fig 3, S3 Fig, S4 Fig), and interestingly ILEI overexpression does not modulate any aspects of phenotype switching (S5 Fig). We should note that we cannot distinguish whether the ILEI overexpression results are due to the limitations of the experimental conditions or because of biological realities. This finding that ILEI contributes to partial phenotype switching is important because therapeutic targeting of phenotype switching will require the fine dissection of its multiple molecular components. For instance in the case of ILEI, one could imagine a therapeutic scenario in which inhibition of ILEI would shift the cells from an invasive state to an intermediate state with decreased metastatic potential and an unchanged proliferative rate whereas a broad inhibitor of the invasive state would shift the cells to a proliferative state with decreased metastatic potential and increased proliferative rate.

Specifically with regards to ILEI we offer the following discussion. We believe melanoma could serve as a useful model to illuminate the molecular details of ILEI. First of all, ILEI expression increases during melanoma progression (Fig 1). Second, within melanoma cell lines we found that ILEI expression correlates with the cell’s invasive phenotype (Fig 2). Third, we found that ILEI expression can be changed by phenotype switch between the MITF-high proliferative and the MITF-low invasive phenotype (Fig 3). Finally, we found that modulation of ILEI affects invasive potential in MITF-low invasive melanoma cell lines. In sum, melanoma gives us a cell model in which ILEI expression is related to the cell phenotype, and ILEI is contributing to that phenotype.

We believe new models could benefit the ILEI field, considering that the molecular details of ILEI-induced EMT have been elusive [55]. The receptor for ILEI is still unknown, and though it has been described to activate STAT and ERK signaling, the evidence is inconsistent. ILEI undergoes post-translational processing, and it seems that the cellular context is critical for the elucidation of details relevant to ILEI [59]. Therefore, given a clear role for ILEI in our ILEI-high MITF-low cells, we conducted gene expression analyses hoping to garner clues towards the molecular mechanism of ILEI-induced invasion (Fig 5). We were intrigued to see AKT/mTOR in the gene set enrichment analysis, and we will conduct future studies to delineate ILEI signaling pathways. A second intriguing hit from our gene expression analysis is interferon response. This caught our attention for two reasons. The first is technical; shRNA gene expression analyses are commonly fraught with the non-specific activation of viral response genes [86]. This occurs because shRNA is double stranded RNA, which activates Toll-like receptors to induce interferons [87]. While our experiments included controls including scrambled shRNA, three different shRNA sequences targeting ILEI, and two different cell lines, we cannot rule out the possibility of a non-specific response. A second reason we noticed interferon response in the gene set enrichment analysis is because ILEI is a secreted cytokine-like molecule, and while the evidence described in this manuscript suggests that ILEI plays an autocrine tumor cell function, an alluring alternative is that ILEI can act in a paracrine manner to regulate the tumor infiltrating immune system. Future studies will explore the role of ILEI in the immune system.

One final finding from this paper that requires further reflection is the regulation of ILEI expression. To date there are three known regulators of ILEI: the first is translational regulation of ILEI by TGF-β/AKT2/hnRNP-E1, the second is degradation of ILEI by the ubiquitin/proteasome system, and the third is an autophagy-mediated increase in ILEI protein expression [49, 61, 62]. Based on the data presented herein, we believe none of these three mechanisms are responsible for regulation of ILEI in the melanoma cell line model (S2 Fig). First, translational regulation of ILEI by TGF-β/AKT2/hnRNP-E1 was originally described in a breast cancer model and we did not observe any evidence of AKT-mediated regulation of ILEI expression (S2 Fig), and while we observed an effect of TGF-β on ILEI expression this was at the mRNA level (Figs 1–3 and data not shown). Second, degradation of ILEI by the ubiquitin/proteasome system was originally described in a prostate cancer model and we did not observe any effect of MG-132-mediated proteasome inhibition on ILEI expression in the melanoma cell line model (S2 Fig). Finally, in regards to the autophagy-mediated increase in ILEI expression, this was described in a melanoma cell line model identical to the one in this study [61]. However, we believe our observations in this study suggest a novel mechanism of ILEI regulation at the transcriptional level, and this will be the subject of future studies.

To conclude, we show here that phenotype switching in melanoma regulates ILEI expression, and knockdown of ILEI attenuates invasive potential in MITF-low invasive melanoma cells, but does not affect chemoresistance or MITF expression.

## Supporting information

S1 Fig Validation of ILEI antibody on ILEI knockdown cell lines (ab72182, rabbit α ILEI). **A.** Immunoblot analysis of ILEI-low 501-Mel cells expressing shSCR, shILEI 3, or shILEI 5.
(TIFF)

S2 Fig The relationship between PTEN, autophagy, and the ubiquitin/proteasome system with ILEI expression. **A.** Immunoblot analysis of PTEN and AKT levels in WM3912, WM983, 501-Mel, Sk-Mel-28, WM793, 1205Lu, and WM9 cells. **B.** Immunoblot analysis of PTEN, AKT, and ILEI levels in ILEI-low WM3912 stably transduced pools with pLKO.1-puro scrambled shRNA or two different shRNAs specific for PTEN. **C.** Immunoblot analysis of LC3B and ILEI levels in ILEI-low 501-Mel or ILEI high 1205Lu cells treated with chloroquine (lysosomal inhibitor, 100 μM, 1h). I and II indicate LC3B pre-lipidation or post-lipidation, respectively. **D.** Immunoblot analysis of ubiquitin and ILEI levels in ILEI-low WM983B or ILEI-high 1205Lu cells treated with MG-132 (proteasomal inhibitor, 10 μM, 0 to 2h).
(TIFF)

S3 Fig The effect of ILEI knockdown in ILEI-high/MITF-low cells on chemoresistance. **A.** Immunoblot analysis of ERK and BIM levels in 501-Mel or 1205Lu cells treated with vemurafenib (BRAFi, 24h, 1 μM). EL indicates the extra-long isoform of BIM. **B.** MTT analysis of WM3912, WM983B, 501-Mel, Sk-Mel-28, WM793, or 1205Lu cells treated with vemurafenib (0 up to 10 μM, 4d). Solid lines indicate ILEI low cells and dashed lines indicate ILEI high cells. **C.** Clonogenic survival assay of WM3912 and WM793 cells treated with vemurafenib (10 μM, 4d). **D.** Immunoblot analysis of ERK, ILEI, and BIM levels in ILEI-high 1205Lu cells expressing shSCR or shILEI 3. Cells were treated with vemurafenib (24h; 1 μM). EL indicates extra-long isoform of BIM. **E.** MTT analysis of 1205Lu cells expressing shSCR or shILEI 3 treated with vemurafenib (72h, 0 up to 50 μM). Solid lines indicate shSCR and dashed lines indicate shILEI 3. N = 3, mean +/- SD, * indicates p < 0.05 by unpaired Student’s t-test. **F.** Clonogenic survival assay of 1205Lu cells expressing shSCR or shILEI 3 treated with vemurafenib (7 days, 1 μM). Images are representative of five independently seeded experiments. **G.** FACS analysis of 1205Lu expressing scrambled shRNA or shILEI 3 treated with vemurafenib (48h, 5 μM). Black bars indicate Annexin V-FITC low and PI low cells, light grey bars indicate Annexin V-FITC low and PI high cells, dark grey bars indicate Annexin V-FITC high and PI low cells, and white bars indicate Annexin V-FITC high and PI high cells. N = 3, mean +/- SD, n.s. indicates p > 0.05 by unpaired Student’s t-test.
(TIFF)

S4 Fig The effect of acquired vemurafenib resistance on ILEI expression. **A.** Immunoblot analysis of ERK, BIM, and ILEI in parental Sk-Mel-28 or WM983B cells or those with acquired vemurafenib (BRAFi) resistance were treated with vemurafenib (24h, 0 up to 5 μM).
(TIFF)

S5 Fig The effect of ILEI overexpression in ILEI-low/MITF-high cells on proliferation, migration, and chemoresistance. **A.** Immunoblot analysis of ILEI and V5 of ILEI-low Sk-Mel-28 or 501-Mel cells stably overexpressing a C terminal V5-tagged ILEI construct. **B.** Cell counts of Sk-Mel-28 or 501-Mel cells expressing vector or ILEI. Solid lines indicate vector and dotted lines indicate ILEI. Data is representative of two independently seeded replicates. **C.** Wound healing assay of Sk-Mel-28 or 501-Mel cells expressing vector or ILEI. Images shown are representative of three independently seeded replicates. **D.** Quantification of panel C using ImageJ software. N = 3, mean +/- SEM, and * indicates p < 0.05 by unpaired Student’s t-test. Black bars indicate vector and white bars indicate ILEI. **E.** Immunoblot analysis of ERK, ILEI, and BIM levels in 501-Mel cells stably transduced with vector or ILEI. Cells were treated with vemurafenib (24h; 1 μM). EL indicates extra-long isoform of BIM. **F.** MTT analysis of 501-Mel cells expressing vector or ILEI treated with vemurafenib (72h, 0 up to 50 μM). Solid lines indicate vector and dotted lines indicate ILE. N = 3, mean +/- SD, * indicates p < 0.05 by unpaired Student’s t-test.
(TIFF)

S1 Table sh and siRNA sequences. (DOCX)

S2 Table Primer sequences. (DOCX)

S3 Table WM9 and 1205Lu shSCR and shILEI microarray data. (ZIP)
