# Supplementary figures and images for: Interleukin-like EMT inducer regulates partial phenotype switching in MITF-low melanoma cell lines
Source: PLoS One. 2017 May 17;12(5):e0177830. doi: 10.1371/journal.pone.0177830 (PMC5435346; doi:10.1371/journal.pone.0177830)

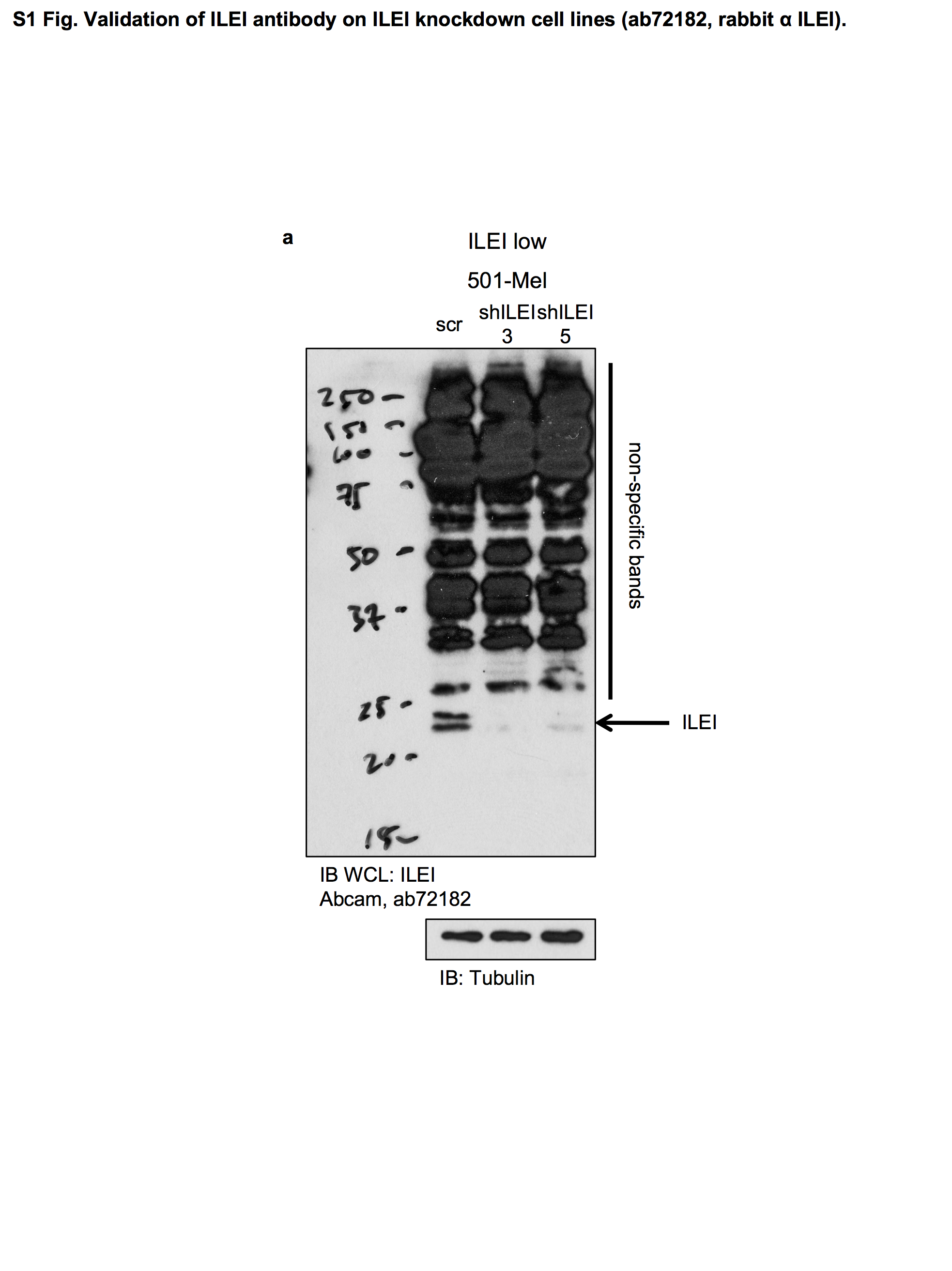

Supplement: S1 Fig — A. Immunoblot analysis of ILEI-low 501-Mel cells expressing shSCR, shILEI 3, or shILEI 5. (TIFF) [file pone.0177830.s001.tiff]

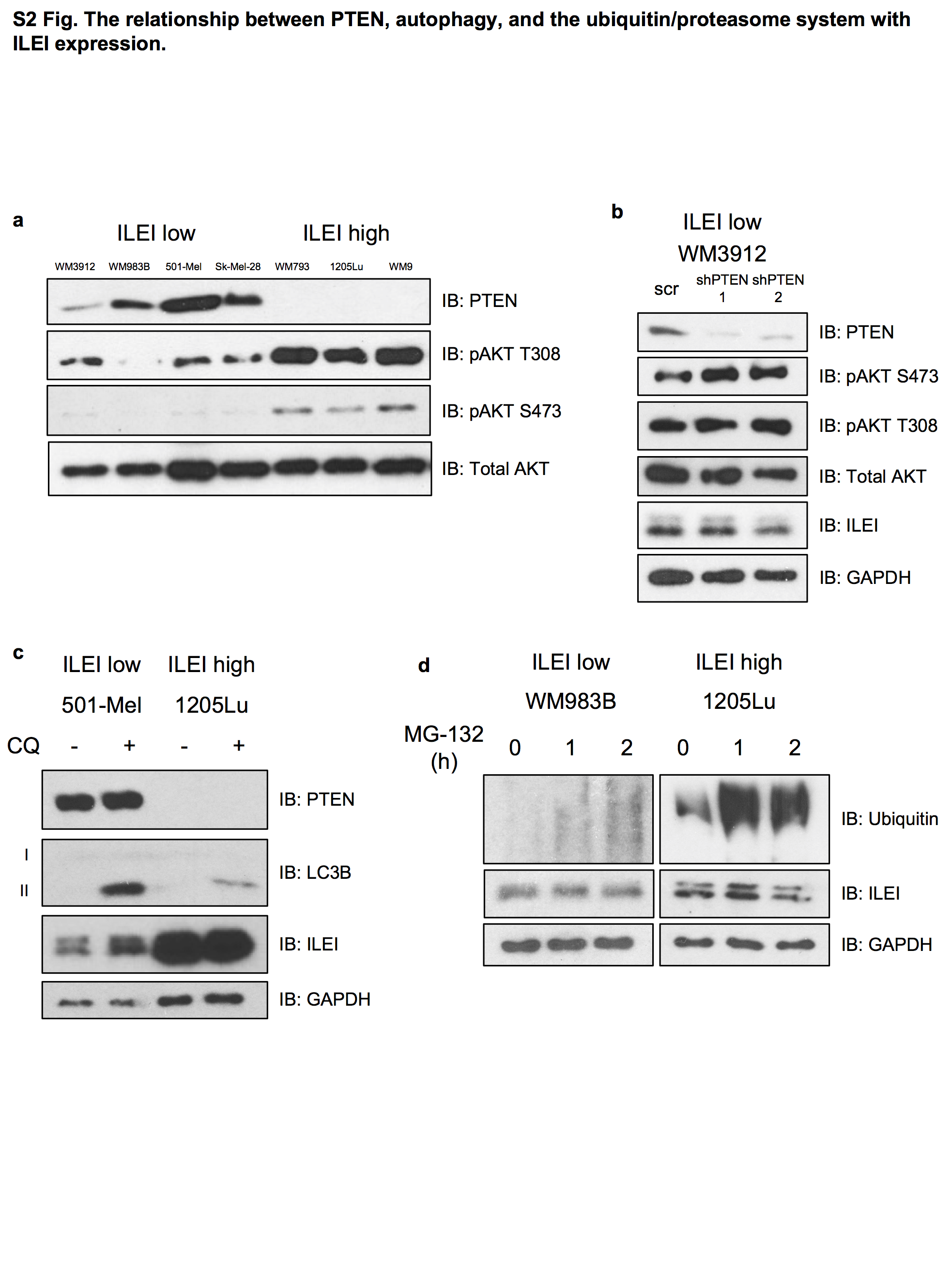

Supplement: S2 Fig — A. Immunoblot analysis of PTEN and AKT levels in WM3912, WM983, 501-Mel, Sk-Mel-28, WM793, 1205Lu, and WM9 cells. B. Immunoblot analysis of PTEN, AKT, and ILEI levels in ILEI-low WM3912 stably transduced pools with pLKO.1-puro scrambled shRNA or two different shRNAs specific for PTEN. C. Immunoblot analysis of LC3B and ILEI levels in ILEI-low 501-Mel or ILEI high 1205Lu cells treated with chloroquine (lysosomal inhibitor, 100 μM, 1h). I and II indicate LC3B pre-lipidation or post-lipidation, respectively. D. Immunoblot analysis of ubiquitin and ILEI levels in ILEI-low WM983B or ILEI-high 1205Lu cells treated with MG-132 (proteasomal inhibitor, 10 μM, 0 to 2h). (TIFF) [file pone.0177830.s002.tiff]

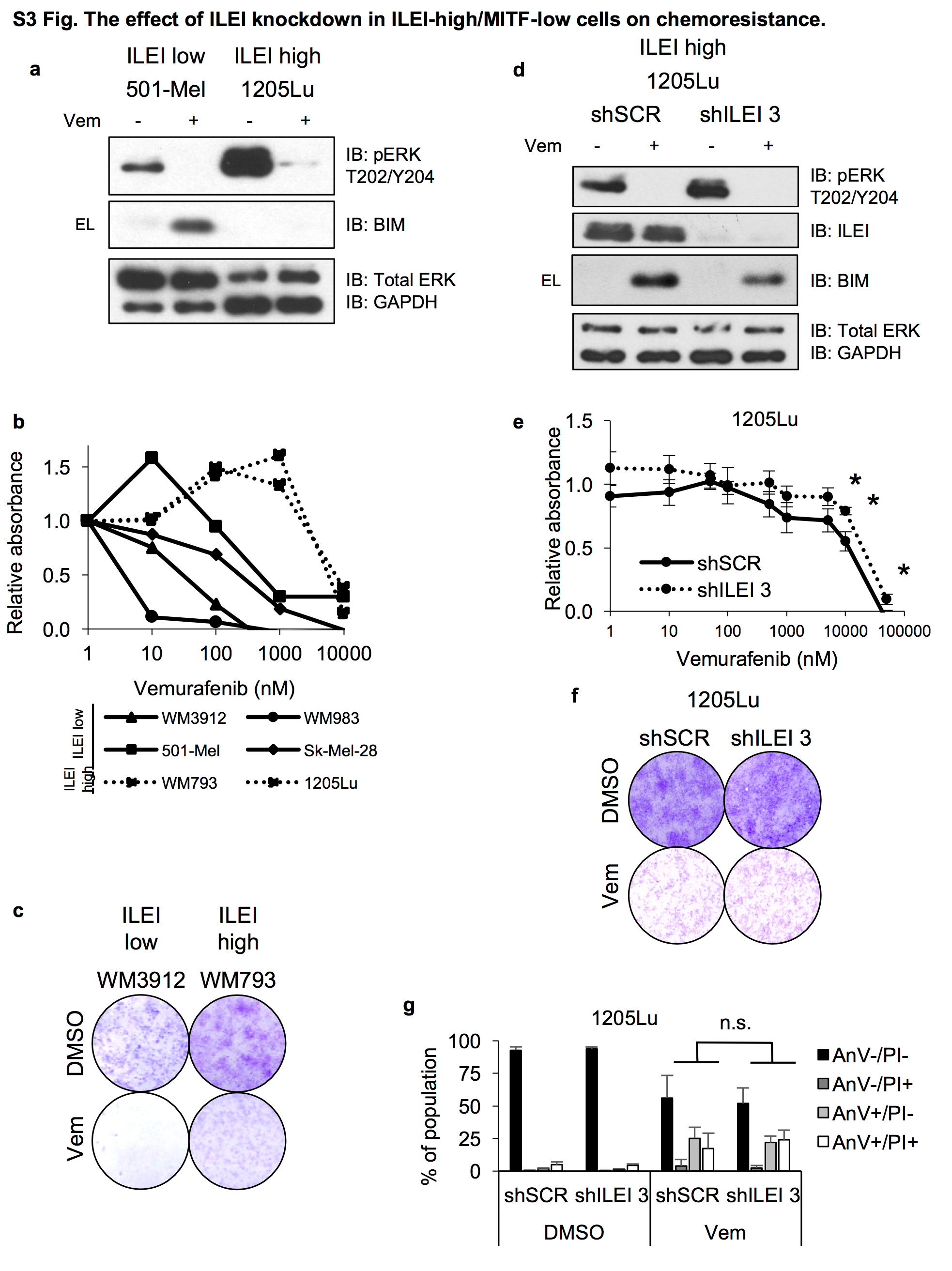

Supplement: S3 Fig — A. Immunoblot analysis of ERK and BIM levels in 501-Mel or 1205Lu cells treated with vemurafenib (BRAFi, 24h, 1 μM). EL indicates the extra-long isoform of BIM. B. MTT analysis of WM3912, WM983B, 501-Mel, Sk-Mel-28, WM793, or 1205Lu cells treated with vemurafenib (0 up to 10 μM, 4d). Solid lines indicate ILEI low cells and dashed lines indicate ILEI high cells. C. Clonogenic survival assay of WM3912 and WM793 cells treated with vemurafenib (10 μM, 4d). D. Immunoblot analysis of ERK, ILEI, and BIM levels in ILEI-high 1205Lu cells expressing shSCR or shILEI 3. Cells were treated with vemurafenib (24h; 1 μM). EL indicates extra-long isoform of BIM. E. MTT analysis of 1205Lu cells expressing shSCR or shILEI 3 treated with vemurafenib (72h, 0 up to 50 μM). Solid lines indicate shSCR and dashed lines indicate shILEI 3. N = 3, mean +/- SD, * indicates p < 0.05 by unpaired Student’s t-test. F. Clonogenic survival assay of 1205Lu cells expressing shSCR or shILEI 3 treated with vemurafenib (7 days, 1 μM). Images are representative of five independently seeded experiments. G. FACS analysis of 1205Lu expressing scrambled shRNA or shILEI 3 treated with vemurafenib (48h, 5 μM). Black bars indicate Annexin V-FITC low and PI low cells, light grey bars indicate Annexin V-FITC low and PI high cells, dark grey bars indicate Annexin V-FITC high and PI low cells, and white bars indicate Annexin V-FITC high and PI high cells. N = 3, mean +/- SD, n.s. indicates p > 0.05 by unpaired Student’s t-test. (TIFF) [file pone.0177830.s003.tiff]

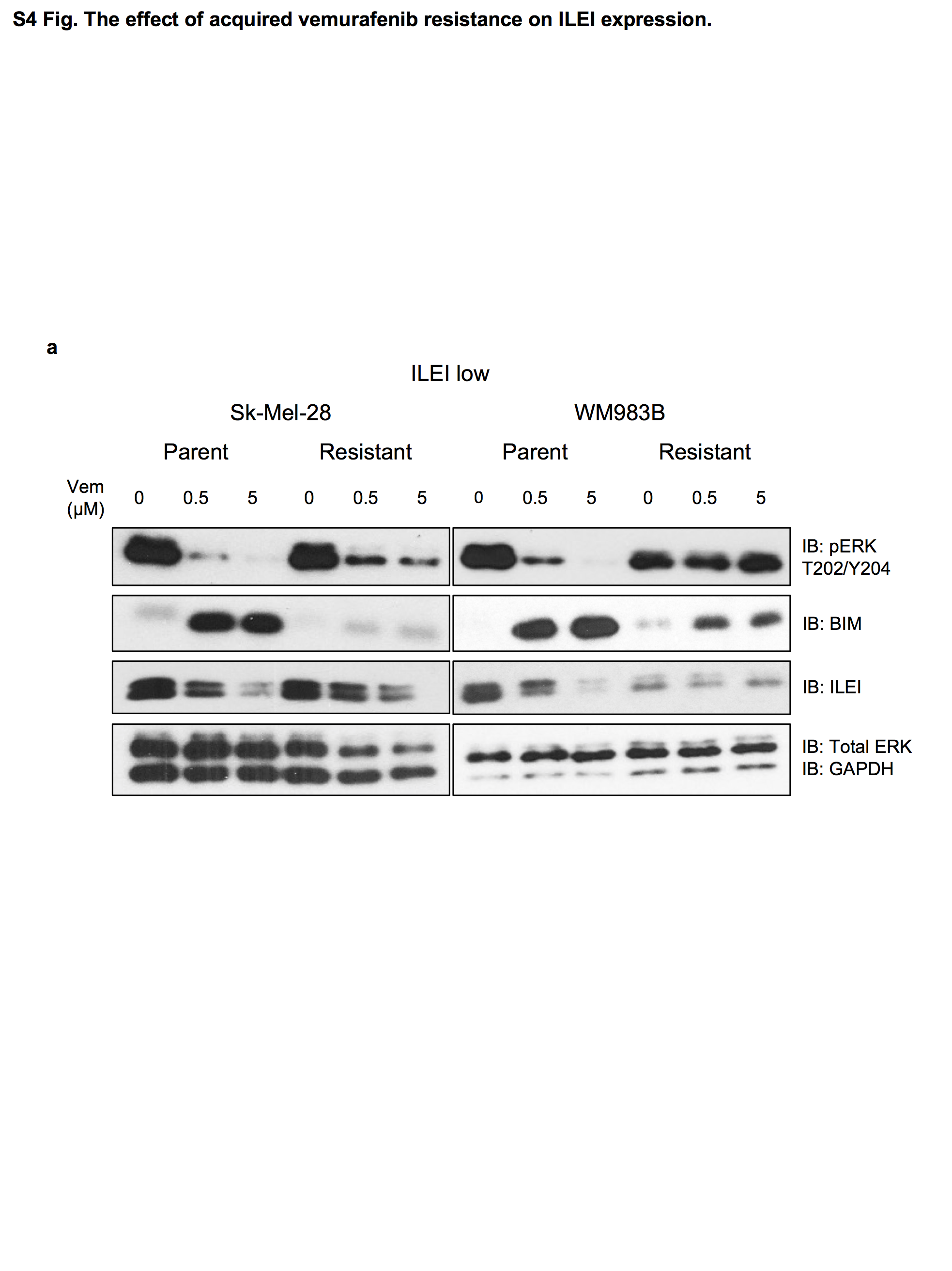

Supplement: S4 Fig — A. Immunoblot analysis of ERK, BIM, and ILEI in parental Sk-Mel-28 or WM983B cells or those with acquired vemurafenib (BRAFi) resistance were treated with vemurafenib (24h, 0 up to 5 μM). (TIFF) [file pone.0177830.s004.tiff]

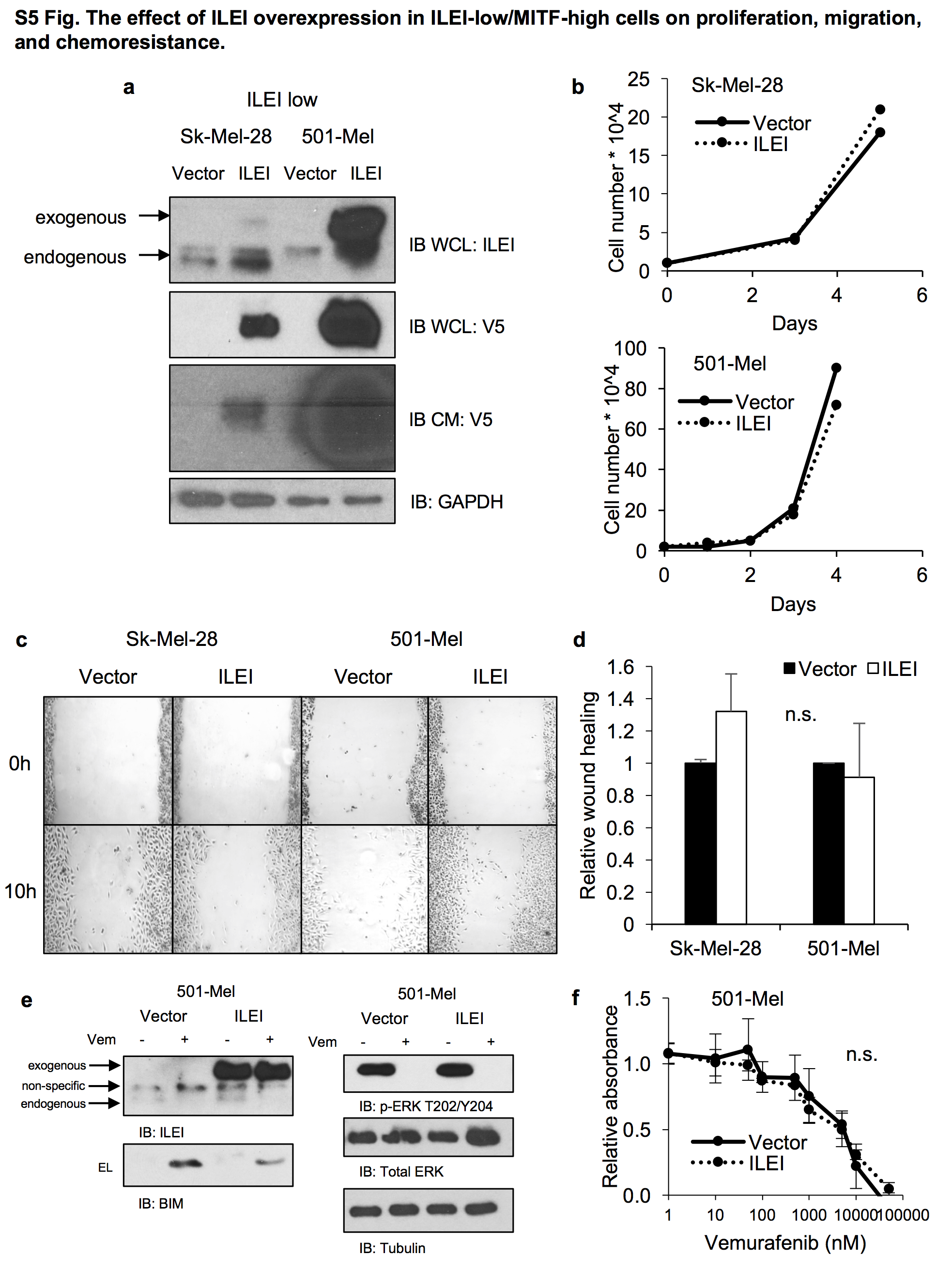

Supplement: S5 Fig — A. Immunoblot analysis of ILEI and V5 of ILEI-low Sk-Mel-28 or 501-Mel cells stably overexpressing a C terminal V5-tagged ILEI construct. B. Cell counts of Sk-Mel-28 or 501-Mel cells expressing vector or ILEI. Solid lines indicate vector and dotted lines indicate ILEI. Data is representative of two independently seeded replicates. C. Wound healing assay of Sk-Mel-28 or 501-Mel cells expressing vector or ILEI. Images shown are representative of three independently seeded replicates. D. Quantification of panel C using ImageJ software. N = 3, mean +/- SEM, and * indicates p < 0.05 by unpaired Student’s t-test. Black bars indicate vector and white bars indicate ILEI. E. Immunoblot analysis of ERK, ILEI, and BIM levels in 501-Mel cells stably transduced with vector or ILEI. Cells were treated with vemurafenib (24h; 1 μM). EL indicates extra-long isoform of BIM. F. MTT analysis of 501-Mel cells expressing vector or ILEI treated with vemurafenib (72h, 0 up to 50 μM). Solid lines indicate vector and dotted lines indicate ILE. N = 3, mean +/- SD, * indicates p < 0.05 by unpaired Student’s t-test. (TIFF) [file pone.0177830.s005.tiff]
