# Supplementary material for: Interleukin-like EMT inducer regulates partial phenotype switching in MITF-low melanoma cell lines
Source: PLoS One. 2017 May 17;12(5):e0177830. doi: 10.1371/journal.pone.0177830 (PMC5435346; doi:10.1371/journal.pone.0177830)
Supplement: S1 Table — (DOCX) [file pone.0177830.s006.docx]

**S1 Table. sh and siRNA sequences.**

| shRNA and siRNA | Referred to herein as | Sequence | Source |
| --- | --- | --- | --- |
| pLKO.1-puro Non-mammalian shRNA | SCR | CCGGCAACAAGATGAAGAGCACCAACTCGAGTTGGTGCTCTTCATCTTGTTGTTTTT | MUSC Hollings Cancer Center shRNA Shared Resource Technology |
| TRC2-pLKO-puro TRCN0000298583 | shILEI 3 | CCGGATGTTGGAAGAGGGATCAATGCTCGAGCATTGATCCCTCTTCCAACATTTTTTG | MUSC Hollings Cancer Center shRNA Shared Resource Technology |
| TRC2-pLKO-puro TRCN0000298584 | shILEI 4 | CCGGGAGGAGATGTGGCACCATTTACTCGAGTAAATGGTGCCACATCTCCTCTTTTTG | MUSC Hollings Cancer Center shRNA Shared Resource Technology |
| TRC2-pLKO-puro TRCN0000293815 | shILEI 5 | CCGGCTTGGTGTGTGCATGAGTATTCTCGAGAATACTCATGCACACACCAAGTTTTTG | MUSC Hollings Cancer Center shRNA Shared Resource Technology |
| TRC2-pLKO-puro  TRCN0000329863 | shMITF 1 | CCGGCTGCACTGCATTCGCACAAACCTCGAGGTTTGTGCGAATGCAGTGCAGTTTTTG | MUSC Hollings Cancer Center shRNA Shared Resource Technology |
| TRC2-pLKO-puro  TRCN0000329793 | shMITF 2 | CCGGCGGGAAACTTGATTGATCTTTCTCGAGAAAGATCAATCAAGTTTCCCGTTTTTG | MUSC Hollings Cancer Center shRNA Shared Resource Technology |
| TRC2-pLKO-puro  TRCM0000019123 | shMITF 3 | CCGGCGGGAAACTTGATTGATCTTTCTCGAGAAAGATCAATCAAGTTTCCCGTTTTT | MUSC Hollings Cancer Center shRNA Shared Resource Technology |
| TRC2-pLKO-puro  TRCN0000002747 | shPTEN 1 | CCGGCTAGAACTTATCAAACCCTTTCTCGAGAAAGGGTTTGATAAGTTCTAGTTTTT | MUSC Hollings Cancer Center shRNA Shared Resource Technology |
| TRC2-pLKO-puro  TRCN0000002749 | shPTEN 2 | CCGGCCACAAATGAAGGGATATAAACTCGAGTTTATATCCCTTCATTTGTGGTTTTT | MUSC Hollings Cancer Center shRNA Shared Resource Technology |
| SignalSilence® PTEN siRNA I #6251 | siPTEN | - | Cell Signaling Technology |
| SignalSilence® Control siRNA (Unconjugated) #6568 | siCTRL | - | Cell Signaling Technology |
