# Supplementary material for: Interleukin-like EMT inducer regulates partial phenotype switching in MITF-low melanoma cell lines
Source: PLoS One. 2017 May 17;12(5):e0177830. doi: 10.1371/journal.pone.0177830 (PMC5435346; doi:10.1371/journal.pone.0177830)
Supplement: S2 Table — (DOCX) [file pone.0177830.s007.docx]

**S2 Table. Primer sequences.**

| Primer name | Sequence |
| --- | --- |
| RT-PCR ACTB F | ATG CTT CTA GGC GGA CTA TG |
| RT-PCR ACTB R | ACA AAT AAA GCC ATG CCA AT |
| RT-PCR DCT F | CTA AGG AGG GAG GGA GAG GG |
| RT-PCR DCT R | GGA TTT TGC AGC CCA AGC AA |
| RT-PCR FAM3C F | GCA ACC AAA CTC AAT GAT GA |
| RT-PCR FAM3C R | ACC ACA GAA GAC CCA GTT GT |
| RT-PCR GAPDH F | CTC CTC ACA GTT GCC ATG TA |
| RT-PCR GAPDH R | GGT TGA GCA CAG GGT ACT TT |
| RT-PCR MITF F | CTT AAA AGC ATC CGT GGA CT |
| RT-PCR MITF R | ACC AAA TCT GGA GAG CAG AG |
| RT-PCR ZEB1 F | GGC GCA ATA ACG GAA AGG AAG |
| RT-PCR ZEB1 R | TGA GGA GAA CTG GTT GCC TG |
| RT-PCR ZEB2 F | CAG CTG AGG TTA TGG CTC CC |
| RT-PCR ZEB2 R | CAA CCC TGA AAC AGA AGG CCC |
